# “*Better a one-dimensional image than no image at all*” – an interview study on nursing educators’ views on patient-perspective simulations

**DOI:** 10.1186/s12912-026-04397-2

**Published:** 2026-02-06

**Authors:** Anna Christine Steinacker, Michael Klingenberg, Lukas Bischof, Marion Diegelmann, Stefan Bösner

**Affiliations:** 1 Department of Health Sciences, University of Applied Sciences Fulda, Leipzigerstr. 123, 36037 Fulda, Germany; 2 School of Medicine, Philipps University Marburg, Biegenstraße 10, 35037 Marburg, Germany; 3 Goethe University Frankfurt, University Hospital Frankfurt, Theodor-Stern-Kai 7, 60590 Frankfurt am Main, Germany

**Keywords:** Patient-perspective, Experience-based learning, Simulation-based learning, Point of view, Nursing education

## Abstract

**Background:**

Patient-perspective simulations are frequently implemented in nursing education to help students reflect on the lived realities of those they will care for. However, little is known about how educators who design and facilitate these exercises perceive and implement them.

**Methods:**

We conducted 16 semi-structured interviews with nursing educators from eight nursing schools in Germany, covering both vocational and academic programmes. Data were analysed using grounded theory methodology to explore educators’ perspectives on the purpose, implementation, and perceived impact of patient-perspective simulations.

**Results:**

Through the process of coding, three themes with subthemes were identified. Theme 1 *Educational purposes of patient-perspective simulations* highlights educators’ views on the value of these exercises, what their aims are, making learning enjoyable, and creating relevant experiences. Theme 2 *Creating an environment of trust* demonstrates that psychological safety and group dynamics are important. Theme 3 *“It’s approximation – not imitation*” reflects educators’ awareness that such exercises cannot replicate patients’ lived experiences completely, but can serve as valuable entry points for reflection if appropriately guided and debriefed.

**Conclusion:**

Overall, educators regarded patient-perspective simulations as an effective means of encouraging reflection, and greater sensitivity to the patients’ experiences, while also acknowledging their limitations. Educators play an important role in framing and guiding these exercises to prevent oversimplification, manage group dynamics, and create meaningful learning.

**Supplementary Information:**

The online version contains supplementary material available at 10.1186/s12912-026-04397-2.

## Introduction

Professional nursing care requires a collaborative and holistic approach that takes into account the unique needs of each individual receiving care [1]. To meet this objective, nursing students must acquire both theoretical and procedural knowledge as well as the ability to critically reflect on their actions and decisions [2, 3]. Nursing is an inherently relational and complex profession, requiring practitioners to engage with multiple perspectives, amongst others; their own, those of patients, and those of families [4, 5]. The ability to see the world through another’s eyes is often seen as central to developing empathy [6], a quality widely regarded as crucial in nursing education and practice [7].

Patient-centered competencies are explicitly emphasized in the national nursing training guidelines in Germany, which recommend the use of experiential and simulation-based methods [8]. Experiential learning places immediate personal experience at the centre of the educational process. Active involvement of learners bridges abstract concepts with concrete experiences, thus ensuring a deeper and more meaningful understanding of the content [9]. So-called *patient-perspective simulations* have been integrated into curricula of both academic and vocational nursing schools. Patient-perspective simulations, also referred to as point-of-view simulations or disability simulations, are designed to simulate physical and emotional aspects of illness, disability or other conditions that temporarily limit mobility or independence [10, 11]. They include activities such as lying in a hospital bed [12], maneuvering a wheelchair [13], wearing a simulated ostomy [14], experiencing auditory hallucinations through earphones [15], wearing an age suit [16], obesity suit [17], or pregnancy belly [18], simulation of living with specific illnesses e.g. diabetes [19] or experiencing sensory loss [20]. Virtual and augmented reality (VR/AR) applications are also being used to facilitate patient-perspective simulations. One example is the *Virtual Dementia Tour*, which enables participants to experience the world from the perspective of a person living with dementia [21]. These exercises are intended to deepen students’ understanding of patients’ experiences and the emotional, physical, and social dimensions of care [22] by placing learners in situations of vulnerability and dependency [11, 23]. Additionally, they are supposed to promote empathy and reflection to prepare them for the complexity of professional nursing care [11].

Recent reviews highlight both potentials and limitations of these methods. A systematic review found that simulations requiring learners to act in the role of a patient may be particularly effective in fostering empathy [23]. Similarly, a scoping review concluded that emotionally and bodily engaging simulations can strengthen empathy, although their long-term effects remain uncertain [11]. At the same time, several studies have pointed to important limitations. By focusing primarily on technical aspects, these exercises might be unable to capture everyday realities faced by people with disabilities, potentially oversimplifying or misrepresenting their lived experiences [10, 24]. Some authors further caution that such activities can unintentionally reinforce stereotypes or evoke negative emotional responses, as confronting simulated dependency may provoke discomfort, fear, or insecurity among learners [11, 20].

While many studies examine how students perceive and benefit from patient-perspective simulations [14, 17, 25], comparatively little attention has been given to the role of educators in shaping and guiding these experiences. As designers and facilitators of experiential learning, educators have valuable insights into the pedagogical goals, challenges, and potential of such exercises. Understanding their perspectives is key to developing meaningful and effective teaching practices that bridge the gap between theoretical instruction and empathetic, patient-centered care. Although this study is situated within the German nursing education context, patient-perspective simulations are widely used in nursing and health professions education internationally. Similar exercises have been described across different countries and educational systems, making questions of pedagogical framing, ethical considerations, and educator responsibility relevant beyond national contexts. Exploring educators’ perspectives can therefore contribute to broader discussions on experiential learning and empathy development in nursing education.

This study therefore addresses the following research questions,

- Which types of patient-perspective simulations do participating nursing educators report using in their teaching practice?
- Why do nursing educators implement these exercises, and what educational goals do they aim to achieve through them?
- What key conditions and considerations do educators identify as important for the successful implementation of patient-perspective simulations?

## Methods

### Participants and setting

This study adopts a qualitative approach to explore educators’ perspectives on the implementation and impact of patient-perspective simulations in nursing education. Nursing educators, clinical mentors/teachers, and skills lab tutors were recruited from eight nursing schools in Germany, including both academic and vocational programs. In Germany, nursing education is organized through two main pathways, a vocational route or an academic route leading to a bachelor’s degree in nursing. While the vocational pathway represents the predominant form of nursing education nationally [26], both pathways incorporate simulation-based and experiential teaching methods, including patient-perspective simulations [8]. The inclusion of educators from both settings was intended to capture a range of pedagogical contexts rather than to reflect the national distribution of nursing students. Participants were recruited using purposive sampling through the research team’s professional networks and direct contact with nursing schools known to implement patient-perspective simulations. In most cases, initial contact was made with the head of the nursing department, who provided information about the study and identified educators who met the inclusion criteria. Eligible educators were then contacted directly by the researcher, received written study information, and were invited to participate on a voluntary basis. Eligibility was confirmed during initial contact and again prior to the interview.

Inclusion criteria were (1) currently working as an educator in a nursing school, (2) regular implementation of patient-perspective simulations in teaching (at least once per semester), and (3) provision of informed consent.

A total of 16 educators participated in the study. All were registered nurses through the vocational or the academic programme. Professional roles included nurse educators from academic (*n* = 6) and vocational (*n* = 8) schools or both (*n* = 1), and one skills lab tutor. The majority had completed additional qualifications; 13 held a Master’s degree and 2 held a Bachelor’s degree (see Table 1).

**Table 1:** Participants’ characteristics

Participants	Total	16
Age	25–30	3
	30–40	6
	40–50	4
	50 +	3
Years active in Education	Under 5 years	3
	5–10 years	7
	10–20 years	4
	Over 20 years	2
Gender	Female	12
	Male	4
Role(s)*	Nurse Educator (academic)	6
	Nurse Educator (vocational)	8
	Nurse Educator (both)	1
	Skills Lab Tutor	1
Highest degree	Master’s Degree Nurse Education	7
	Other Master’s Degree (General Education, Public Health, APN or Nursing Science)	6
	Bachelor’s Degree (Nursing Education and Nursing Management)	2
	Vocational Nursing Degree	1

Recruitment and data collection proceeded concurrently and continued until thematic saturation was reached, defined as the point at which no substantially new themes emerged from successive interviews. After 16 interviews, additional data no longer contributed new conceptual insights, and recruitment was therefore concluded.

### Data collection

Data were collected through focused interviews according to Merton, a semi-structured method that invites participants to reflect in depth on concrete teaching situations [27]. An interview guide with open-ended questions was specifically developed for this study by the research team, which includes expertise in nursing education, nursing practice, and qualitative methods. The guide addressed educators’ experiences with patient-perspective simulations, perceived educational goals, challenges encountered, and pedagogical framing of these activities. An English translation of the full interview guide is provided as Supplementary Material 1.

Interviews were conducted between January and August 2025 by the first author (AS). Interviews lasted between 25 and 65 min and were primarily conducted in person. Four interviews were conducted online to accommodate participants’ availability. All interviews were audio-recorded with participants’ consent, conducted in German, and transcribed verbatim according to the guidelines described by Kuckartz [28]. For publication purposes, all quotations were translated into English by the primary author, with careful attention to preserving meaning and tone.

### Data analysis

Data were analyzed using coding procedures derived from grounded theory methodology, as originally described by Glaser and Strauss [29], which were applied as analytical tools rather than for theory generation. Analysis began immediately after the first interview, allowing emerging insights to inform subsequent interviews [30].

The first stage involved open coding, during which the primary author AS and LB examined each transcript line by line to identify key concepts. During this phase, MK and SB were regularly consulted to discuss emerging codes and interpretations. In vivo coding was used to preserve participants’ original language, and memo-writing was employed to document emerging thoughts and analytical reflections. Constant comparison was applied throughout this phase to ensure rigor and consistency [30]. In the subsequent axial coding phase, preliminary codes were discussed, refined, and grouped into higher-order themes during collaborative group analysis sessions involving AS, MD, LB, and MK. These sessions focused on comparing interpretations, clarifying relationships between codes, and jointly developing subthemes and thematic linkages. Finally, the main themes that structured the findings were agreed upon through team discussion and consensus. MAXQDA software was used to support the systematic organization of the data and the coding process.

### Ethical considerations

The study received ethical approval from the Ethics Committee of the School of Medicine at the University of Marburg. All participants provided informed consent after receiving written and oral information. Confidentiality and ethical standards were maintained throughout the process. The study was conducted in accordance with the ethical principles of the Declaration of Helsinki. All data were anonymized, and identifying details were removed from transcripts and reports to protect participants’ privacy.

## Results

### Implemented exercises

The participating educators described a wide range of exercises designed to engender perspective-taking. Table 2 provides an overview of specific activities the participants regularly use as well as how many of the participants reported them. The exercises named ranged from basic care activities (washing, oral care, being assisted with eating, moved, or placed on a bedpan) to somatic simulations (wearing incontinence materials, a colostomy bag, obesity or age suits, tremor simulation). Several educators also used mobility or sensory restrictions (wheelchair use, visual impairment, physical restraints), while a smaller number included clinical procedures.

**Table 2:** Exercises used by interview partners (IP)

Exercises to provoke Perspective-Taking	
Receiving a bed bath / being washed	10
Receiving oral care	3
Being assisted with eating / feeding	7
Experiencing visual impairment or blindness (e.g., through eye masks)	5
Experiencing physical restraints	1
Being repositioned or transferred / manual handling	6
Wearing incontinence materials	6
Using a bedpan	4
Wearing a simulated colostomy bag	1
Navigating with a wheelchair or mobility aid	5
Transportation in a hospital bed	2
Wearing a simulated obesity suit	3
Wearing an age simulation suit	6
Placement of naso-gastric tube	3
Experiencing tremor (via simulation gloves)	1

### Themes

Qualitative analysis revealed three primary themes each capturing central aspects of educators’ perspectives on patient-perspective simulations. Each theme was further differentiated into three subthemes (see Table 3).

**Table 3:** Main themes and subthemes

Theme	Subthemes
Theme 1 Educational purposes of patient-perspective simulations	*Patient-perspective simulations are essential*
	*The exercises are often fun*
	*The experiences stay with you*
Theme 2 Creating an environment of trust	*It’s a respectful*,* protective space*
	*No random group assignment*
	*Always keep an eye on the groups*
Theme 3 It’s approximation – not imitation	*It’s a bit like cultural appropriation of Illness*
	*Better a one-dimensional image*,* than no image at all*
	*Educators as guides to reflection*

## Theme 1 Educational purposes of patient-perspective simulations

Educators highlighted several reasons for using patient-perspective simulations in nursing education. They saw these exercises as a way to improve reflective thinking, provide engaging and enjoyable learning experiences, and create lasting impressions that help students remember and apply what they have learned during encounters with patients.

### Patient-perspective simulations are essential

There was clear consensus among interviewed educators that these exercises are essential to nursing education. Several stressed that they should be a standard part of every programme, *“Absolutely. I’d definitely say they belong in every training programme.”* Educators also highlighted their value for promoting empathy and understanding, *“It’s about developing understanding*,* showing empathic behavior*,* being able to put yourself in someone else’s shoes… that’s extremely important.”* They further noted the potential for self-reflection, *“As their studies progress*,* students themselves become more aware of this and increasingly self-reflective.”* Finally, the exercises were seen as shaping professional attitudes, *“I’m confident it will change how I act at the patient’s bedside and how I approach someone.”*

### The exercises are often fun

Interviewees emphasized that experience-based exercises were valued not only for their pedagogical impact but also for the sense of enjoyment they created. As one participant explained, these activities offered students a welcome break from routine, *“It’s nice to escape a little from the usual classroom setup—sitting in front of the tablet*,* staring at the front*,* and just working through tasks.”* Educators themselves often enjoyed the exercises, *“Yes*,* I really liked doing it*,* because it’s always a bit of fun.”* Beyond personal enjoyment, they emphasized that fun was intentionally built into the design to support learning, *“The idea is also that students should have fun with it—it should be enjoyable for them.”* Fun was not regarded as trivial, but as a key motivational factor, *“Yes*,* I’d definitely say it’s often fun […] enjoying learning is important*,* because the more fun you have*,* the more interest you show in the topic.”*

### The experiences stay with you

Educators suggested that patient-perspective simulations likely have a lasting impact on students, drawing both on their own experiences and on what they observed in learners. They described these activities as creating memorable moments that stayed with students more vividly than traditional lectures. As one participant noted, *“Definitely*,* it has a lasting effect. With an exercise like this*,* you remember it much more clearly than any lecture in day X.”* Others reflected on how the exercises shaped their own perspective, *“I have to say*,* it really opened my eyes to how patients perceive things.”* Educators also highlighted the strong personal impression these exercises left on them, underlining their value beyond immediate teaching outcomes, *“That was so impressive that I carried it with me*,*”* and *“I can still remember it really well.”*

## Theme 2 Creating an environment of trust

Across all interviews, educators reported the importance of trusting each other within the group during patient-perspective exercises. Educators described strategies for guiding a safe and supportive environment, including creating a respectful and protective space, allowing students to choose their own partners or smaller groups, and closely observing the interactions to ensure comfort and boundaries are respected.

### *It’s a respectful*,* protective space*

Educators reported that trust within the group is a prerequisite for patient-perspective exercises to succeed. Because these activities often involve vulnerability, dependence, or even embarrassment, students need to feel safe in order to participate meaningfully. As one participant explained, *“If the group’s trust isn’t there*,* then they just wouldn’t do it… otherwise*,* it’s well accepted.”* The level of trust was described as varying greatly across groups, shaping both participation and engagement, *“It really depends on the group and the level of trust within it… I’ve had groups where it didn’t go so well*,* and others where it went very differently.”* Creating a protected space with respectful interactions was therefore seen as a central responsibility of educators. As one interviewee put it, *“Everyone has to understand it’s a protected space and I have to feel okay with the people in the small group I’m working with.”* Ultimately, trust was not considered merely helpful but essential to fostering reflective, experiential learning. As several participants emphasized, it is the foundation that allows students to engage openly and transform uncomfortable moments into meaningful learning opportunities.

### No random group assignment

Closely linked to the importance of trust, many participants emphasized that students should be allowed to choose their own partners for patient-perspective simulations. Granting this autonomy was described as a key condition for creating the psychological safety needed for such activities to be effective. As one educator explained, *“Another important point is that I never assign groups randomly*,* like counting off numbers. I let them choose partners themselves. That’s very important in my view.”* Others reinforced this perspective, stressing the positive effects of self-selection, *“They were allowed to assign themselves”* and *“I’m basically interested in the groups assigning themselves.”* Allowing students to work with peers they trust was seen as essential not only for engagement but also for ensuring that the experience is remembered positively, *“For me*,* it’s absolutely crucial that students leave this […] learning space feeling secure*,* not dreading the next time they’re grouped with someone randomly and going in with a bad mood.”*

### Always keep an eye on the group

Interviewees emphasized that educators play a central role in monitoring and shaping group dynamics during patient-perspective simulations. One participant stressed the need for close attention, *“You need to be very attentive*,* that’s why you should know the group well.”* Such attentiveness enables educators to recognize early signs of discomfort or exclusion and to respond appropriately. Rather than limiting their role to logistical organization, educators described themselves as facilitators of emotional safety. As one participant explained, it is essential to, *“always keep an eye on the group—how are they handling it? Or individuals*,* too”*, particularly when exercises address emotionally or physically sensitive issues.

## Theme 3 *It’s an approximation – not imitation*

A recurring thread in the interviews was the ambivalence surrounding how much patient-perspective simulations can actually convey the realities of patients or affected individuals. While participants consistently acknowledged that these simulations cannot provide an authentic understanding of another person’s lived experience, they also defended the relevance of such exercises for their promoting awareness and critical reflection.

### It’s a bit like cultural appropriation of illness

Participants agreed that patient-perspective exercises should be understood as entry points rather than full replications of patients’ lived experiences. Educators consistently emphasized that such simulations can only provide a simplified or partial impression of reality. As one reflected, *“We looked at one perspective and thought*,* ‘So this is what it feels like.’ But that might be wrong. You might think sitting on a bedpan isn’t that bad — but none of us has had a femur fracture. So did we really experience it at all?”* One participant captured this gap in particularly striking terms, *“It’s like pretending to be sick*,* almost like cultural appropriation of illness.”* Others echoed this account of simplification, *“It’s definitely a simplified image”* and *“Patient-perspective exercises are not reality.”* Ultimately, educators highlighted that these exercises should not be mistaken for authentic patient experiences, but rather valued for their potential to start reflection and empathy when appropriately framed.

### *Better a one-dimensional image*,* than no image at all*

Interviewees emphasized that the purpose of patient-perspective simulations is not to achieve realism, but to foster emotional and cognitive engagement. As one participant explained, *“Even if it’s not realistic — better a one-dimensional image than no image at all.”* Several described the exercises as valuable entry points, even if they only provide a partial glimpse into patients’ lived realities, *“As a first step*,* as an impulse*,* it’s definitely more meaningful than doing nothing at all”*, and *“I only have the chance to briefly step into that world of experience.”* The central value, educators argued, lies in the exercises’ potential to trigger reflection, *“If students or trainees at least get that initial nudge to think about how they communicate—what words they use*,* or how they explain medical or nursing-related matters—then that’s already a big win*,* if it gets them to reflect and think critically.”* Another participant summarized, *“I don’t think the goal is to fully place yourself in someone else’s position. The goal is much more to prompt someone to engage deeply with the situation.”*

### Educators as guides to reflection

Participants stressed that for patient-perspective simulations to be truly meaningful, educators must carefully frame and guide the experience, both before and after the activity. One educator emphasized the importance of clarifying the limitations from the outset, *“Patient-perspective simulations are not reality. That’s something I need to make clear right at the start.”* Another highlighted the critical role of structured reflection, *“That’s where educators come in — to guide the reflection*,* how exhausting was that? What if you had to live with this constantly? What would it mean for the patient?”* Educators noted that debriefing often reveals how differently individuals interpret the same exercise, “*the reflection afterward is often where you realize just how differently people perceive the same experience.*” Some suggested involving affected persons in the design or evaluation of exercises to enhance authenticity and ethical sensitivity, “*It would be interesting to ask affected people how they perceive these exercises — maybe even involve them in one.*”

## Discussion

The aim of this study was to explore nursing educators’ perspectives on patient-perspective exercises, focusing on the implementation, considerations of importance, and attitudes. Among the German nursing educators interviewed in this study, commonly used patient-perspective simulations included personal care, such as bed baths, hair washing, and oral hygiene. Incontinence care was also common, particularly through exercises where students wore disposable briefs or experienced sitting on a bedpan. Simulation suits, most often the age-simulation suits, were widely used, as were exercises related to mobility and sensory loss. Notably, none of the participants in this study reported using virtual or augmented reality (VR/AR) systems for patient-perspective exercises.

In the process of coding, three core themes were identified; Theme 1, *Educational purposes of patient-perspective simulations*, Theme 2, *Creating an environment of trust*, and Theme 3, *It’s approximation – not imitation.*

The first theme, “Educational purposes of patient perspective simulations” is explained by three central features (subthemes); they carry meaning, they are enjoyable, and they have an impact on practice. All interviewees viewed patient-perspective simulations as an important tool for helping students understand the perspectives of patients and family members, while shaping attitudes, perspectives, and empathy toward patients. These findings align with previous studies, which have primarily focused on short-term effects on learners [12, 14, 16, 25], while leaving questions about sustained or long-term impacts largely unexamined. Enjoyment emerged as another important component. As highlighted in prior research on these types of exercises, fun can enhance learning processes [31], and participants in our study similarly emphasized the importance of the enjoyment of the learning method. This interpretation aligns with experiential learning theories, which emphasize that learning is deepened when learners are actively involved and emotionally engaged [9].The perceived influence of patient-perspective exercises was often discussed in relation to the educators’ own experiences. Many interviewees shared personal anecdotes from their own training and emphasized how richly they remembered such exercises, leading them to assume that similar experiences would leave a lasting impression on their students as well. While this suggests a potential long-term effect, educators in this sample acknowledged that they have no direct evidence of lasting changes in students’ attitudes or behaviors. This aligns with the earlier studies on patient-perspective simulations, which identify a gap in longitudinal research. Only a few studies followed-up with learners after the initial exercise, for example, one follow-up in a study conducted by Basit et al. reported no long-term impact [32], while another by Chaffin & Adams described behavioral changes, but only based on unsystematic observations in clinical practice without a systematic evaluation [33].

The second theme, “Creating an environment of trust” shows the importance that the educators placed on trust as foundational for patient-perspective simulations. Because these exercises often place students in positions of dependence, vulnerability, or even embarrassment, the social context in which they occur strongly shapes how they are experienced. As Goffman argued in *The Presentation of Self in Everyday Life* [34], every social encounter carries the risk of deep embarrassment, particularly when one’s usual presentation of competence is disrupted. Patient-perspective simulations heighten this risk, since students must *play* a dependent role that contrasts with the professional identity they are in the process of developing [23].

In this context, psychological safety becomes crucial. Edmondson defines psychological safety as “a shared belief that the team is safe for interpersonal risk taking” [35]. Without such safety, students may resist engaging fully in the exercise or may experience it as threatening rather than transformative. This is especially relevant in simulation-based learning formats or role-play, where psychological safety has been shown to directly influence learning outcomes [36].

The interviews further highlighted that trust is not only a matter of group atmosphere, but also deeply connected to the professional conduct and competencies of educators. As van Dyk et al. [37] emphasize, trust is facilitated by the ethical behavior, knowledge, and skills of those who design and facilitate the learning environment. This aligns with participants’ views that educators bear responsibility for ensuring an atmosphere of safety, guiding the process, and debriefing experiences in ways that transform potentially embarrassing or unsettling moments into meaningful learning opportunities [23].

The third theme, “It’s approximation, not imitation” looked at how much these simulations can imitate the lived experiences of patients. Our participants repeatedly emphasized that simulations can never reproduce the individual experience of illness or disability, but that they remain “*better than nothing*” in instigating reflection and deeper engagement with patients’ situations. This position contrasts with some of the literature, which offers pointed criticism. Disability simulations have been described as providing *“totally false impressions”* [24], evoking pity [38], or reinforcing stereotypes of incompetence and dependency [10, 20]. Participants in earlier studies even reported feeling relieved not to be disabled or embarrassed that they were “mocking” disabled people [39, 40]. Such responses risk trivializing disability or misrepresenting its realities, especially given that simulated impairments are temporary and escapable [24]. While this critique is valid, the educators in our study approached the issue differently. They did not expect the exercises to reproduce real experiences in full. Instead, they saw the value in using them as teaching tools to instigate reflection, encourage empathy, and create room for dialogue. As several interviewees explained, even a simplified or “one-dimensional” experience can still be worthwhile if it gets students thinking about how they communicate and care for patients. For educators, this pragmatic view made sense, since true replication is impossible, the focus shifts to what the exercises can realistically achieve in the classroom. This resonates with perspectives suggesting that while simulations cannot authentically replicate disability, they can serve as embodied entry points into perspective-taking, provided they are framed critically and paired with guided reflection [11, 41, 42]. To further reduce misrepresentation and increase authenticity, educators suggested involving service users, such as patients or family members, in designing, facilitating, and debriefing exercises. This aligns with research that emphasizes the value of co-production and service user involvement in health professions education [20, 31, 42].

It should also be noted that much of the critique of disability simulations comes from research contexts outside of nursing education. While these concerns remain valid, in nursing education the pedagogical aim is less about reproducing disability experience and more about sensitizing students to the patient-perspective as a foundation for their daily clinical practice. To make this possible, our participants emphasized that educators play a crucial role in framing, guiding, and debriefing such exercises to prevent misinterpretations and create meaningful learning [11, 23].

### Strengths and limitations

This study is the first to explore patient-perspective simulations from the standpoint of German educators, offering valuable insights into how these practices are implemented, perceived, and justified in nursing education. However, the sample of educators in this study does not represent German nursing education. While most nursing students in Germany pursue the vocational training route, almost half of the interviewees in this study were from academic institutions. As a result, the perspectives of educators from vocational settings may be underrepresented, which limits the transferability of the findings to the broader German nursing education system.

The convenience sampling used in the study also provided a limitation. Study participants were voluntarily recruited through professional networks, which may have provided self-selection bias, as educators who volunteered were likely positive and supportive of patient perspective simulations, meaning that more critical perspectives remain underexplored.

Finally, the study is limited by its reliance on self-reported accounts, which capture beliefs and experiences but not the actual classroom dynamics or long-term impact on students. Observational or longitudinal research could complement these findings by examining how patient-perspective simulations are enacted in practice and how they influence learners over time.

### Implications for educators

Based on our findings, several practical implications can be drawn for the implementation of patient-perspective simulations.

- Be mindful of group composition; ensure psychological safety by allowing students to work with peers they feel comfortable with.
- Frame exercises carefully; emphasize that they are not “real” experiences of illness or disability but approximations designed to trigger reflection.
- Integrate real patient-perspectives; use reflective written accounts by patients, narratives, or involvement of service users to enhance authenticity.
- Always debrief; provide structured feedback and reflection after the exercise to connect the experience with professional learning.

## Conclusion

This interview study provides insight into educators’ ideas and thoughts on patient-perspective simulations. All participating educators expressed strong support for using these methods, stressing their value in helping students to gain insight into the experiences of others, provided that a safe learning environment and structured reflection are ensured.

Future research should examine whether and how collaboration with service users can contribute to the design and implementation of these exercises. In addition, more studies are needed to explore the long-term effects of patient-perspective simulations on students’ empathy, professional attitudes, and care practices.

## Supplementary Information

Below is the link to the electronic supplementary material.

Supplementary Material 1

## Data Availability

The analysed data for this study is not publicly available, to preserve anonymity and integrity of the participants but is available on reasonable request via the corresponding author.
