# Supplementary material for: “Better a one-dimensional image than no image at all” – an interview study on nursing educators’ views on patient-perspective simulations
Source: BMC Nurs. 2026 Feb 6;25:220. doi: 10.1186/s12912-026-04397-2 (PMC12977884; doi:10.1186/s12912-026-04397-2)
Supplement: Supplementary file 1 — Supplementary Material 1 [file 12912_2026_4397_MOESM1_ESM.docx]

**Supplementary Material 1**

**“*Better a One-Dimensional Image than no Image at All*” – An Interview Study on Nursing Educators’ Views on Patient-Perspective Simulations**

**Anna C. Steinacker, Michael Klingenberg, Lukas Bischof, Marion Diegelmann and Stefan Bösner**

**Interview Guide**

**Introductory Statement**

“Thank you very much for taking the time to participate in this interview.
As mentioned in the invitation, the purpose of this conversation is to explore your experiences, perspectives, and methodological approaches regarding *patient-perspective simulations* in nursing education.

With your permission, I will make an audio recording of this interview so that I can transcribe your responses accurately. The recording and transcript will be handled confidentially and used solely for research purposes. Your participation is voluntary, and you may withdraw at any time without any negative consequences.

Before we begin, do you have any questions about the interview or the use of the data?

If you agree, I will now start the recording.”

**Main Interview Questions**

**1. Understanding and Definition**

**Main Question:**

- What comes to your mind when you think of *patient-perspective simulations*?

**2. Reactions of Trainees or Students**

**Main Question:**

- How do trainees or students generally respond to *patient-perspective simulations*?

**Sub-questions (if not covered):**

- Could you share examples where reactions were particularly strong or noticeable?
- How do you handle criticism or reservations from students?
- Are there situations in which you would consciously decide not to use such simulations? Why?

**3. Design and Implementation**

**Main Question:**

- How do you select or design the *patient-perspective simulations* that you use in your teaching?

**Sub-questions (if not covered):**

- Could you describe the development and implementation of one specific simulation, including goals, preparation, and follow-up?
- What challenges or obstacles do you encounter during the preparation or implementation phase?

**4. Reflection and Follow-up**

**Main Question:**

- How do you structure the follow-up phase after a simulation?

**Sub-questions (if not covered):**

- Do you use specific reflection methods or models (e.g., structured feedback rounds, reflective journals, plenary discussions)?
- How do students typically engage in these reflective processes?

**5. Effects and Perceived Benefits**

**Main Question:**

- To what extent do *patient-perspective simulations* contribute to the development of professional competencies in nursing students?

**Sub-questions (if not covered):**

- What changes or learning outcomes have you observed following these simulations?
- What limitations or weaknesses do you perceive in using *patient-perspective simulations* in nursing education?

**6. Closing Question**

**Main Question:**

- Is there anything important to you that we have not yet discussed?

**Closing Remark**

“Thank you very much for your time and for sharing your experiences.
Your insights are extremely valuable for understanding how *patient-perspective simulations* are used in nursing education. If you wish, I can send you a short summary of the study results once the research has been completed.”
